# Enhancing Monte Carlo Tree Search for Retrosynthesis

**DOI:** 10.1021/acs.jcim.5c00417

**Published:** 2025-06-13

**Authors:** Ton M. Blackshaw, Joseph C. Davies, Kristian T. Spoerer, Jonathan D. Hirst

**Affiliations:** † School of Chemistry, University of Nottingham, University Park, Nottingham NG7 2RD, U.K.; ‡ School of Computer Science, 6123 University of Nottingham, Jubilee Campus, Nottingham NG8 1BB, U.K.

## Abstract

Computer-Assisted Synthesis Programs are increasingly employed by organic chemists. Often, these tools combine neural networks for policy prediction with heuristic search algorithms. We propose two novel enhancements, which we call eUCT and dUCT, to the Monte Carlo tree search (MCTS) algorithm. The enhancements were deployed in AiZynthFinder and have been integrated into the open-source electronic lab notebook, AI4Green, available at https://ai4green.app. A memory-efficient stock file was used to reduce the computational carbon footprint. Both enhancements significantly reduced, by up to 50%, the computational clock-time to solve 1500 heavy (500–800 Da) molecules. The dUCT enhancement increased the number of routes found per molecule for the 1500 heavy molecules and a 50,000-molecule set from ChEMBL. eUCT and dUCT-v2 solved between 600 and 900 more molecules than the unenhanced MCTS algorithm across the 50,000 molecules. When limited to a 150 s time constraint, dUCT-v1 solved ∼5 million more routes to the 50,000 targets than the unenhanced algorithm.

## Graphical abstract

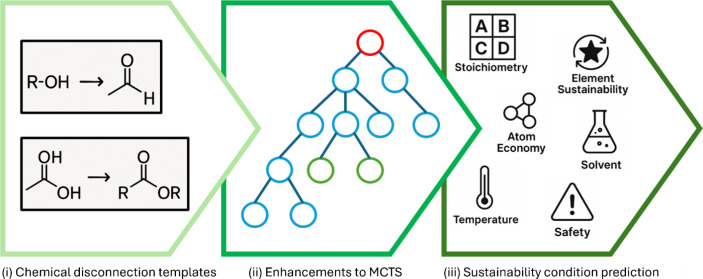

## Introduction

Synthesis planning is the process by which either a computer or chemist advises on how to synthesize a specified compound or ‘target’ molecule. Retrosynthetic analysis is the reverse of this process, where a target molecule is iteratively disconnected into smaller precursors. The sequence of disconnections is then reversed in order, forming a ‘route’ to the target molecule. This method was pioneered by Corey et al.^1,2^ and was predominantly performed manually by organic chemists.^3^ Corey also established the Logic and Heuristics Applied to Synthetic Analysis (LHASA) principles, many of which are still used by chemists and Computer-Assisted Synthesis Planning (CASP) programs.^4^ During the recent machine learning boom, the use of CASP tools has become increasingly prominent.^5^ As machine learning becomes more useful for retrosynthesis, finding more accurate models, databases, and heuristic algorithms is vital to provide reliability in computer-assisted routes. In this paper, we present some algorithmic enhancements to one of the more promising approaches to be applied to CASP in recent years, namely Monte Carlo Tree Search (MCTS), motivated by the success that related enhancements have had in other problem settings.

There are clear analogies between synthesis planning and deterministic games. Machine learning achievements, such as IBM Deep Blue’s success against World Chess Champion Garry Kasparov in 1997,^6^ have inspired similar implementations for synthesis planning.^7–9^ MCTS is a heuristic search algorithm,^10^ which is built upon the Upper Confidence bounds applied to Trees (UCT) equation^11^ and previous work in the field.^12–14^ Segler et al.’s 3N-MCTS for retrosynthesis^8^ combines three neural networks with MCTS to produce reaction routes. One network is a deep learning model, trained on 12.4 million single-step reactions from the Reaxys database,^15^ to predict the outcome of potential reactions. A filter policy is used as the second network to predict whether the suggested single-step reactions are feasible. The third network utilizes symbolic AI to apply predefined chemical rules derived from retrosynthetic principles to map out viable synthetic routes. This ensures the suggested synthetic steps are chemically valid. The 3N-MCTS method was one of the first that produced routes that were preferred to those found in the literature. It improved the number of solved molecules (95% of 497 diverse molecules) and the search time (by ∼70%) compared with a Best First Search (BFS) method.

Similar strategies to that of 3N-MCTS have been adopted by many other models, for example, Ring Breaker^9^ and AiZynthFinder.^7^ AiZynthFinder is the basis for our work. It is open source, utilizes MCTS, and uses an expansion policy similar to that of 3N-MCTS. AiZynthFinder employs an extended multilayer perceptron called a deep highway network with over 100 hidden layers. Rules were extracted from reaction data from the U.S. Patent and Trademark Office (USPTO).^16^ AiZynthTrain comprises two pipelines.^17^ The reaction data pipeline involves extracting the data (from USPTO), followed by cleaning and validating the reaction SMILES. RDKit is used to sanitize molecules;^18^ ones which have incorrect or unrecognized SMILES are discarded. Templates are extracted using the RDChiral package,^19^ and specific logic is used to target ring-forming reactions by augmenting the atoms involved in the reaction with the atoms in the formed ring, along with their respective heteroatoms. The extraction step generates a unique identifier per template, based on its respective fingerprint. A second pipeline is utilized for expansion policy preprocessing and training.

One key difference between AiZynthFinder and 3N-MCTS is the style of the MCTS. 3N-MCTS utilizes 100,000 iterations per molecule, in contrast to 100 used by AiZynthFinder. This disparity highlights the importance of the value network in AiZynthFinder, as the algorithm relies heavily on the network scores to direct the Monte Carlo sampling. Segler et al.’s approach uses optimized neural network architectures and advanced parallel processing, enabling a higher number of iterations by narrowing the search space and focusing on highly probable transformations. In contrast, AiZynthFinder emphasizes robustness and reproducibility, resulting in fewer, more comprehensive iterations per molecule. Therefore, it will be crucial to balance computational expenditure between the value network and MCTS. Our results show that increasing the iteration count is beneficial in solving more molecules, and our enhancements display a large increase in the number of iterations that can be achieved in a given time.

The MCTS algorithm has four main components: Selection, Expansion, Simulation, and Backpropagation. The algorithm will be passed an initial ‘state’ as the root-node, which could refer to a position in a game or any environment that grants a definable reward from decisions. For retrosynthesis, the initial state is the target molecule represented by a SMILES string. This state will be scrutinized such that the tree contains all the possible decisions or child-nodes that could stem from the root. Finding an unexplored node and creating all its possible children is known as expansion. Whenever a node is expanded, the program will perform the simulation function, which performs a random simulation from each child-node down to an end state or finished position. This is achieved by locating possible moves from a position and randomly selecting one until the position can be referred to as terminal. These individual simulations are counted for all nodes and determine the depth for which an MCTS will be trained. Once a terminal position is reached from the simulation, the state will be passed through an evaluation function. The score from the evaluation is stored in the respective child-node from which the simulation began. One visit is also added to the child-node, and the cumulative scores of all children and the total number of visits is stored within the parent-node iteratively back to the root-node. This method of updating the scores and visits to nodes and their respective parents is referred to as backpropagation. Once the simulation and consequent backpropagation is complete, the observed node will return to the root-node. However, as the node has already been expanded, we now must select which route should be tested further. We use the retrieved statistics from the previous random simulations to perform the selection calculation, or the UCT equation,^11^ which balances exploration and exploitation to direct the search (eq 1). Selection keeps occurring until the observed node has not been expanded before, in which case expansion, followed by simulation and backpropagation will occur, after which the observed node will return to the root-node again, with a slightly updated statistical tree.

$${\mathrm{UCT}}_{i}=\frac{W_{i}}{n_{i}}+C\times \sqrt{\frac{\ln(n_{k})}{n_{i}}}$$ 1

In eq 1, *W*_*i*_ is the score from the child-node *i*, *n*_*i*_ is the number of visits to the child-node *i*, *n*_*k*_ is the number of visits to the parent-node *k* and *C* is a parameter. This method will iteratively build statistics for all nodes that produce promising results. Naturally, if the algorithm is set to run for more simulations, the tree will converge to a more accurate decision tree. Once all the simulations are complete, MCTS will return the sequence of moves which yielded the best result from the evaluation function. For many domains, including retrosynthesis, the algorithm can also return a defined number of best solutions.

There is a plethora of enhancements, all targeting specific attributes of the MCTS algorithm. Enhancements can be categorized as domain-specific, or domain-independent. Domain-independent alterations to MCTS should improve the performance of the algorithm regardless of the application. Examples include parallelization, All Moves As First,^20^ a bidirectional variant,^21^ and RAVE,^22^ among others.^23^ In contrast, domain-specific enhancements are tailored to the application of the algorithm, and thus often have more noticeable effects. One domain-specific enhancement is a value network, which stores information about certain positions or states and a respective score. Another is heuristic-based action pruning, which is often used to reduce search spaces by hard-coding rules to eliminate certain actions.

Five MCTS variants have previously been explored for retrosynthesis.^24^ Modified UCT (mUCT), alters the method to save time on the mandatory ‘ergodic’ step in the method, referring to the mandatory exploration of all possible moves from a given state before a deeper or more biased exploration. mUCT with dynamic ‘C’ (mUCT-dc) introduces dynamic tuning of the parameter *C* in the UCT equation. This method addresses the same problem targeted by the enhancements proposed in our work. However, mUCT-dc focuses on adapting the balance between exploitation and exploration based on how under/overexplored certain areas of the tree are. Polynomial Upper Confidence Trees (PUCT) incorporates a policy network term to the selection phase of MCTS, providing a probability distribution of all possible moves from a given state. The final two enhancements were the use of a value network replacing the traditional Monte Carlo rollouts, and bootstrapping which utilizes the outcomes from self-play in the training of the value network.

In other related work, an Experience Guidance Network has been proposed to enhance the traditional MCTS search tree.^25^ It uses a network trained with synthetic ‘experience’ collected during the search process. The method required fewer iterations on average to find a route than the Retro* method,^26^ and the generated routes were of higher quality in terms of route lengths and number of successful routes. Another approach integrated MCTS with A* search for retrosynthetic planning, to leverage the exploratory strength of MCTS with the goal-driven efficiency of A*.^27^ The method applied the rollout capabilities of MCTS into A*, allowing more focused and efficient pathway determination by evaluating future states more accurately. The study reported significant improvements in identifying routes, particularly for natural products.

Our study aims to provide novel algorithmic enhancements to the MCTS used in AiZynthFinder. The metrics used to define improvement are the proportion of solved molecules, the number of solving routes per molecule, the computational clock time to solve a molecule, and the number of iterations completed within a time limit. We also improve memory usage, by employing a memory-efficient stock file.

## Methods

### Enhanced UCT

We propose enhanced UCT (eUCT), a domain-independent adaptation that manipulates the parameter *C* in the UCT equation. Because each template is scored solely on single-step reactions, each ‘move’ is considered independently. Thus, the further the search is from a leaf-node (a terminal node with no children), the lower the probability of reaching the predicted node score is. For example, let us consider a binary search tree, and envisage a shallow node *A* which is a direct child of the root-node. We will assume every node is unvisited and has a predicted score of 0.5 supplied from a value network. This score indicates that half of the subsequent nodes will be successful. For a binary tree, there will be one successful, and one failed node at each decision point. Therefore, if the tree has a terminal depth, *d*, and our current observed node is at depth, *n*, then the probability of attaining a successful route through an arbitrary simulation is 0.5^(*d*–*n*)^. From this, we can deduce that the probability of reaching successful routes through random simulation will increase as *n* approaches *d*. In the unmodified UCT equation, there is no consideration of how close the search is to terminating. The final iteration performs the same UCT calculation as the first iteration. This often causes the later iterations to be wasted, while performing exploration of unvisited nodes, which have a low probability of yielding a successful route. eUCT takes this into consideration and weights the search toward exploitative nodes incrementally, as the search gets closer toward termination. The adaptations to the parameter *C* in the unmodified UCT equation cause the value of *C* to vary continuously from *C* to *C*/1.5, as the current iteration progresses from 0% to 100% of the total iteration count. This alteration reduces the exploration weighting (the second term in the right-hand side of eq 1) as the search progresses. The changes required for eUCT are shown in eq 2.

$${\mathrm{eUCT}}_{i}=\frac{W_{i}}{n_{i}}+eC\times \sqrt{\frac{\ln(n_{k})}{n_{i}}}$$ 2

where e*C* = *C*/(*S*_*n*_ + 1) and *S*_*n*_ is the current iteration number divided by the maximum number of iterations. An example selection decision from eUCT is shown in Figure 1.

**Figure 1:**
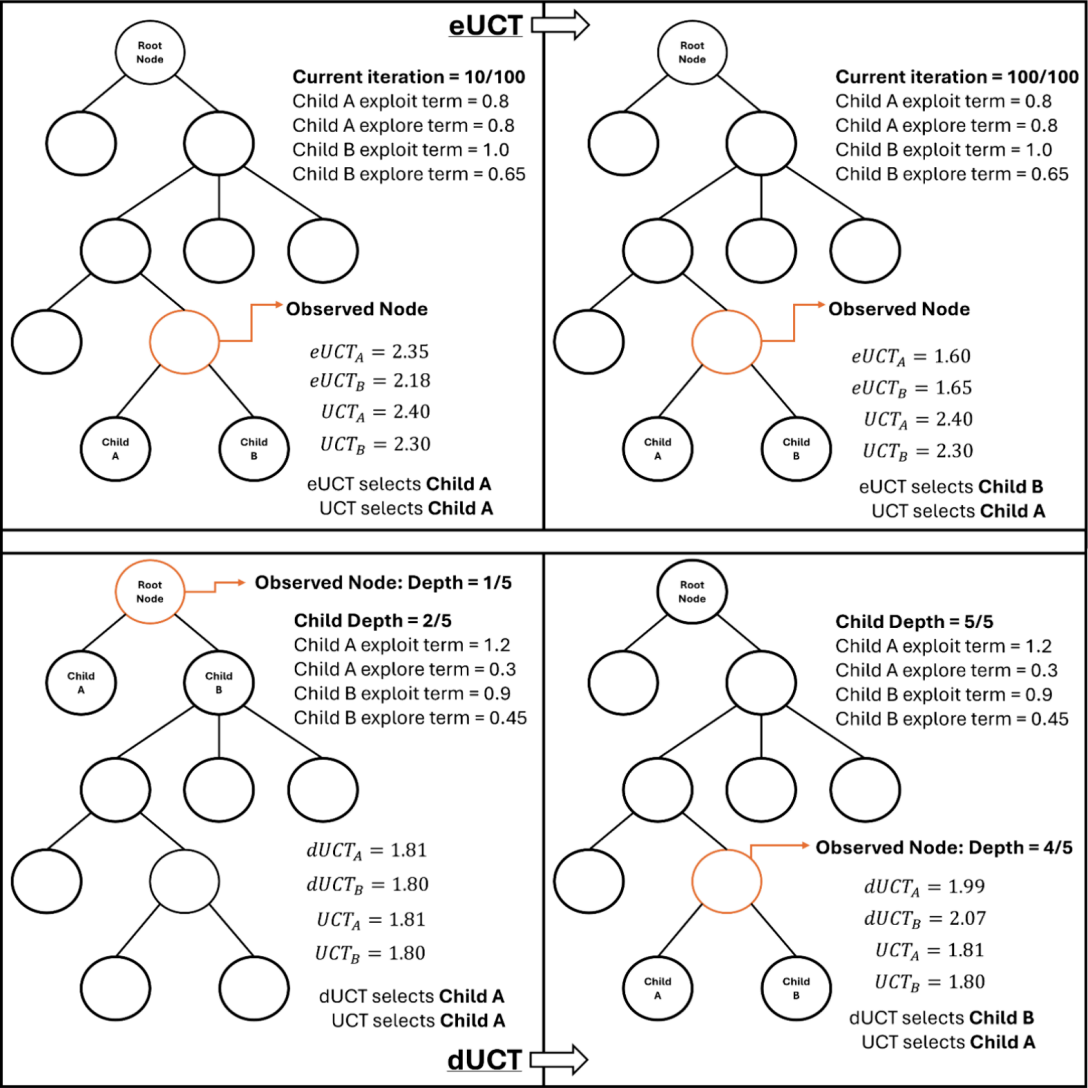
Example scenario of MCTS search tree decision made by eUCT (top row) and dUCT (bottom row), against the default UCT, highlighting the different selection of eUCT at larger iterations, and dUCT at increased depth values.

### Depth UCT

Depth UCT (dUCT), a second enhancement that we propose, is tailored to the problem of retrosynthesis, and is thus domain-specific. Through analysis of retrosynthetic tree structures, we noticed a clear trend. As shown in Figure 2, the branching factor at shallow depths is far larger than when deeper in the tree. Given the increase in routes to explore at earlier stages, it could be more efficient to focus on the high performing routes in shallow depths, as it would be computationally expensive to explore all the possible disconnections of a given molecule when the options are abundant. Therefore, weighting the search toward exploitation while shallow, would reduce exploration of worse nodes. Furthermore, when one gets deeper into the tree, there are fewer available disconnections at each node. We have established from eUCT that the probability of success is higher when deeper in the tree. It would, therefore, be more effective to explore more available disconnections when closer to a leaf-node, as there is a greater probability of locating successful routes.

**Figure 2:**
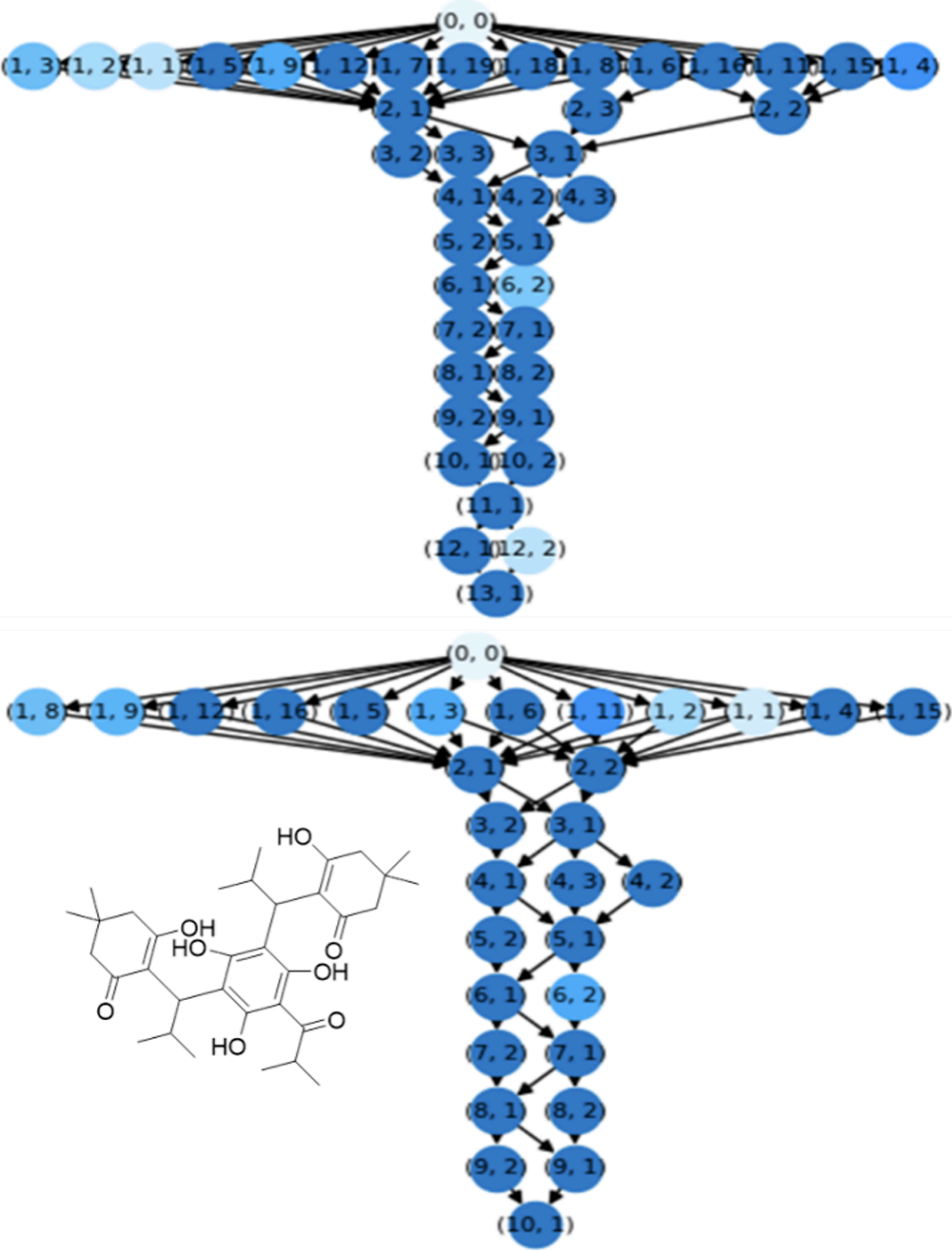
Structure of an example retrosynthesis search tree after 20 iterations for the natural product molecule, C_34_H_48_O_8_, shown inset (31-614-CAS-30049885), which was randomly selected from ChEMBL. The top image is without the enhancement and contains 41 nodes, the bottom image uses the dUCT enhancement and contains 31 nodes. The numbers inside a node correspond to the depth of the node and child-number from the parent-node.

This method is only beneficial due to the predetermined value network in AiZynthFinder. All disconnections have a predicted score, and we utilize this by instantly pruning weak first steps by trusting predictions of the value network and focusing the search on the highest performing first steps. Thus, dUCT is not only domain-specific to retrosynthesis, but reliant on the accuracy of the value network. We implemented this enhancement by passing the depth of the observed node to the dUCT equation (eq 3) and increasing the parameter *C* by an increment, *incr*, multiplied by the current depth, *D*_curr_. The default UCT value is 2, although it can be altered slightly for different domains, so we reduce the starting *C* value to 1.5 in dUCT.

From our preliminary testing, we saw the average depth of search is between four and five. We tested two increments of 0.5 and 0.7 with branching factors of 50 and 20. The method requires manual adaptation of the increment based on the expected largest depth. For example, it would unbalance the equation if one incremented the search by 0.5 per unit of depth if a maximum depth of 50 was expected. Therefore, an understanding of the structural features of the relevant problem is required to implement dUCT, which can be acquired through a program which calculates the average depth of the tree. An example selection decision by dUCT is shown in Figure 1.

$${\mathrm{dUCT}}_{i}=\frac{W_{i}}{n_{i}}+C\times \mathrm{incr}\times D_{\mathrm{curr}}\times \sqrt{\frac{\ln(n_{k})}{n_{i}}}$$ 3

We hypothesized that both enhancements would be more suited to longer multistep syntheses problems, because eUCT adapts to the probability variation in leaf nodes and dUCT bases calculations on node depth. Therefore, one might expect neither enhancement would outperform the default for a single-step synthesis. However, it is worth considering if either enhancement drastically improves the number of iterations per second. Then one can assume more single-step disconnections would be considered. This increase in search space coverage could provide a greater variety of candidate disconnections to the user, although this would not affect finding the algorithm’s perceived ‘best’ disconnections.

### Molecular Data Set

Preliminary testing of the enhancements employed AiZynthFinder version 3.7.^7^ Our initial implementation contained a cutoff number in the configuration, allowing a cap on the number of templates suggested per state, and thus a cap on the branching factor of the search tree. However, most of our tests used version 4.3, after an update to the codebase was released in 2024.^28^ Within this update, the cutoff number was removed to allow for all templates to be eligible for selection if suitable. We reimplemented a branching factor limit, because one of our proposed enhancements requires a strict branching factor.

Genhenden and co-workers tested AiZynthFinder on a set of 497 diverse molecules, against the ASKCOS synthesis planning software.^29^ Comparisons between the two methods proved difficult, due to ill-defined metrics for route comparisons. Thus, our results were initially benchmarked against a 2023 publication that utilized AiZynthFinder to investigate the effects of varied hyper-parameters for multistep synthesis^30^ on a data set of 50,000 (50K) randomly sampled molecules from the ChEMBL database.^31^ For consistency, we used the same data set for primary testing of our enhancements.

For proof-of-concept testing, a smaller set of 1500 ‘heavy’ (between 500 and 800 Da) molecules was sampled from ChEMBL. The performance was analyzed using the following metrics: the proportion of molecules solved for the test set, the absolute number of routes found, the average number of routes found per molecule, and the average computational clock-time per molecule. Both enhancements were run for 100 iterations, with a maximum time limit of 150 s, and a branching factor of 50. For primary testing, AiZynthFinder version 4.3 was used. The literature benchmark for the 50K ChEMBL data set was 73% using the default parameters. We were able to replicate this with AiZynthFinder 3.7. We also tested version 4.3 with the same benchmark data set, with two controls: iteration number and computational clock-time, and we used the ZINC and eMolecules stock files, which contain 17.4 million and 17.6 million compounds, respectively.^32^ The proportion of solved molecules was 76.4%.

As the ZINC^33^ and eMolecules stock files are stored in memory, we created a streamlined MolBloom filter that uses optimized hashing techniques to reduce memory usage and increase the processing capacity for stock file checks.^34^ The bloom filter was trained using 34.5 million unique compounds from eMolecules and ZINC stock. After filtering the stock file, the resultant bloom file was less than 1 GB, which allowed over 20 times as many molecules to be run in parallel. A bloom file is a filter which reduces each item into an array. The array contains *n* items and employs *k* hash functions to map each element to position *k* in the array. The purpose is to predict the position of the observed item, instead of storing the position in memory. This method comes with drawbacks, namely, the filter contains a false positive rate of ∼1%. As our primary objective was to test the enhancements, false positives should not present problems. However, we anticipate a systematic overestimate in the performance metrics for all the algorithmic variants.

### Visualization and Integration within the AI4Green Electronic Lab Notebook

We have integrated CASP tools within the free-to-use and open-source AI4Green electronic lab notebook (ELN).^35^ Embedding a retrosynthesis tool within an ELN obviates the need for a chemist to enter data more than once. Version 4.0 of AiZynthFinder has been integrated into a Flask application. An end point accepts a SMILES string as a target, uses AiZynthFinder to solve the retrosynthesis and returns the solved routes in a JavaScript Object Notation (JSON) format. This method enables the addition of future arguments from AI4Green to the end point, such as user-specified stock lists. The code for the Flask application is stored in a GitHub repository. ASKCOS’s condition prediction tool is a FastAPI application provided by the Machine Learning for Pharmaceutical Discovery and Synthesis Consortium (MLPDS) at MIT on GitLab. Both applications are containerized using Docker. The containers are hosted on Docker Hub and deployed as Azure Container apps.

The Python package Plotly Dash Cytoscape was used to visualize the route data. This displays a collection of connected nodes where each node is a molecule, and the edges linking the nodes denote a retrosynthetic relationship. The Dagre layout arranged the nodes as a treelike structure well suited for retrosynthesis with the target molecule at the top. A nonterminal node will be connected to one or more nodes below, and this iteratively continues until terminal nodes are reached. In chemical terms, a product is connected to one or more reactants below until molecules designated as building blocks by the stock file are reached.

Sustainability metrics are presented, using those from the CHEM21 reaction assessment,^36^ but excluding those (purification method, catalyst recovery, and all yield-based metrics) that cannot be applied before the experiment is carried out. The temperature, solvent, stoichiometry, element sustainability, atom economy, and safety are all assessed based on the predicted route and conditions. Each reaction is individually assessed on these sustainability metrics, and each value is color-coded. Every reaction in a route can also be color-coded by taking a weighted median of the sustainability metrics, where sustainable is equal to 1, problematic 2, hazardous 3, and highly hazardous 4. Each sustainability metric has a corresponding slider that the user can assign between zero and ten; if the weights are all equal, it operates as an ordinary median. The total sum of weights is calculated, and the median or 50th percentile weight is found. The cumulative weights are calculated and the weighted median is the first value where the cumulative weight is greater than or equal to the median obtained in the previous step.

## Results

In our proof-of-concept tests (Table 1) on 1500 heavy molecules, eUCT solved the same number of molecules as UCT, and found ∼0.2 more routes per solved molecule. The low percentage of solved molecules across all methods is due to the complexity of the molecules.

**Table 1:** Testing of Enhancements Using AiZynthFinder 3.7 with the 1500 Heavy Molecule Dataset^a^

method	solve %	total routes	avg search time (s)
UCT	9.5	768	48
eUCT	9.5	794	45
dUCT	11	1041	24

a eUCT and dUCT tested against unmodified UCT for 100 iterations using the ZINC stock.

eUCT is predicated on variation in success probability between explorative nodes and exploitative nodes in the search tree. If there is a lower probability of reaching a successful leaf-node in nodes which have been explored infrequently, which is often the case in MCTS, eUCT should reach successful routes faster in ‘later’ iterations of a search, particularly as they approach the maximum number of iterations. However, AiZynthFinder does not conform to these conditions. The value network provides predictive scores for nodes, regardless of whether they have been visited in the search tree or not. Consequently, if the value network is reliable, there is no difference in the probability of success between deep and shallow nodes because the neural network policy has already predicted the score for each possible disconnection. This could arise from overfitting of the neural network, a speculation that is supported by the repeatability of the results. Repeating a search on the same molecule resulted in near identical solutions, often found during the same iteration. Despite not solving more molecules, eUCT attained a greater number of successful routes, and consequently a higher average route count per solved molecule. A 6% decrease in computational clock-time was observed, which is a statistically significant improvement (a Wilcoxon signed-rank test gave a p-value of 6.9 × 10^–5^). Despite the decrease in clock-time, there was little change in the number of iterations required to solve a molecule. Thus, the speed increase is likely due to fewer nodes being visited per iteration.

More encouragingly, the dUCT enhancement solved more instances in the heavy molecule data set than the unmodified UCT (Table 1). 164 molecules were solved, 15% more than that achieved by UCT and eUCT. dUCT found nearly 300 more routes to target molecules than UCT, a 35% increase. Furthermore, the increase in solving routes is not solely from the increase in the number of solved molecules. dUCT found the highest number of routes per solved molecule: 6.3 compared to eUCT with 5.6 and UCT with 5.4. dUCT improved computational clock-time, by nearly 50% in comparison to UCT. The factors contributing to such improvements are primarily the characteristics of the retrosynthetic search tree. Computational resources are not wasted on early unexplored nodes and are instead focused on exploring productive routes at high depths. The Wilcoxon signed rank test indicated a significant result in favor of dUCT, with a *p*-value of 2.1 × 10^–7^.

Our primary tests (Table 2) on the 50K ChEMBL data set used a combination of the ZINC and eMolecules, and MolBloom stock files. eUCT and two variants of the dUCT enhancement were tested. The first dUCT variant consisted of a 0.7 *C* increment and branching factor of 20, favoring exploration with a lower branching factor. The second used a 0.5 *C* increment with a branching factor of 50. As Table 2 shows, experiments using an iteration-based control yielded a consistent pattern. eUCT displayed minimal change from the unmodified UCT. The solve percentage was identical and a 3% speed increase was observed. This trend was seen for all experiments with eUCT. The performance of the two dUCT variants was different to that seen in the proof-of-concept testing. Unlike the heavy-molecule data set, dUCT-v1 solved a lower number of molecules than UCT, and dUCT-v2 solved ∼0.1% more molecules. This is likely due to the difference in complexity between the two data sets. dUCT was developed to search more at high depths of the tree, to target routes which are difficult to solve. The increased total solve percentage (UCT solved 76.4% of the 50K ChEMBL set compared to 9.5% of the heavy molecule set) shows that the heavy-molecule data set is much more challenging. Thus, a significant improvement in computational clock-time was observed for both variants: 57% for dUCT-v1 and 20% for dUCT-v2. dUCT-v2 solved slightly more molecules, in ∼20% less computational clock-time, with on average 0.6 more routes to a solved molecule than UCT.

**Table 2:** Primary Testing of Enhancements Using AiZynthFinder 4.3 with the 50K ChEMBL Dataset^a^

method	number of iterations	stock files	solve %	routes per molecule	avg time per molecule (s)
UCT	100	ZINC EMOL	76.4	37.8	34.2
eUCT	100	ZINC EMOL	76.4	38.0	33.1
dUCT-v1	100	ZINC EMOL	71.8	32.5	14.3
dUCT-v2	100	ZINC EMOL	76.5	38.6	26.4
UCT	100	MolBloom	86.7	31.3	25.5
eUCT	100	MolBloom	86.7	32.5	24.6
dUCT-v1	100	MolBloom	81.6	25.6	11.9
dUCT-v2	100	MolBloom	86.9	32.1	18.6
UCT	1000	MolBloom	97.0	239.2	255.6
eUCT	1000	MolBloom	97.0	242.9	238.7
dUCT-v1	1000	MolBloom	92.7	170.0	99.1
dUCT-v2	1000	MolBloom	97.0	245.1	170.2
UCT	5000	MolBloom	99.1	962.5	1136.2
eUCT	5000	MolBloom	99.1	965.8	1097.9
dUCT-v1	5000	MolBloom	96.5	884.0	643.5
dUCT-v2	5000	MolBloom	99.2	974.4	974.4

a eUCT and two variants of dUCT: v1 and v2, were run against the unmodified UCT for different values of the maximum number of iterations.

Iteration values of 100, 1000, and 5000 were tested using the MolBloom stock file. Naturally, 5000 iterations gave the highest solve rates, e.g., 99.2% with dUCT-v2. dUCT-v1 had lower total solve rates, but gave speed improvements for the three iteration values (100, 1000, 5000) of 52, 61, and 43%, respectively. dUCT-v2 narrowly improved on UCT for all iteration counts, while showing speed improvements of 27, 33, and 14%, respectively. We initially hypothesized that we would observe increasingly greater speed improvements using dUCT, as the iteration count escalates. As the tree gets larger, the number of nodes pruned through dUCT grows exponentially. However, the 5000 iteration experiments did not yield this result. This is due to several thousand molecules reaching the experiment time-limit of 10,000 s, thus removing the iteration value as the stopping point.

The rate-correct score (RCS) metric^37^ was used to assess the speed-accuracy trade-off. RCS is the number of solved molecules divided by the summed search times, *T*_*i*_. dUCT-v1 attained the highest RCS for all iteration values tested (Figure 3). We conclude that 100 iterations are best for RCS.

**Figure 3:**
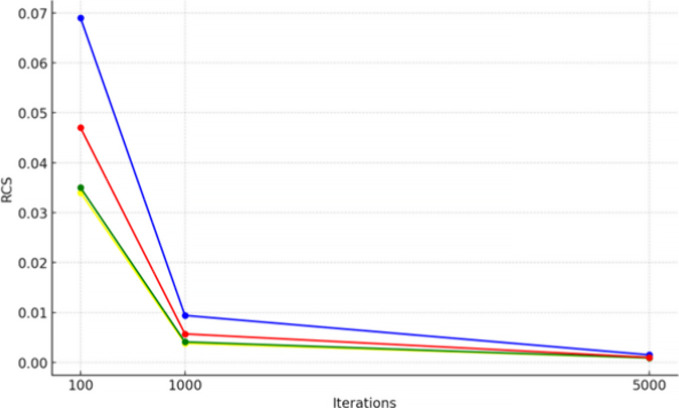
Number of MCTS iterations against the rate correct score (solved molecules per unit of time) for the enhancements eUCT (yellow), dUCT-v1 (blue), dUCT-v2 (red), and the unmodified UCT (green) on the 50k ChEMBL data set using the MolBloom stock.

In time-based control testing (Table 3) both eUCT and dUCT-v2 achieved higher solve percentages than UCT, with increases of 1.2, 1.3, and 0.8% for the respective time constraints (150, 100, and 60 s) for eUCT, and 1.6, 1.5, and 1.8% for dUCT-v2. For the test set of 50,000, this equates to between 400 and 900 more molecules. All enhancements found more routes per molecule than did UCT. dUCT-v1 found the most routes per molecule with improvements of 32.1, 41.0, and 40.2% above UCT for 60, 100, and 150 s, respectively. The latter equates to nearly 5 million more routes across the 50,000 molecules. All enhancements executed more iterations than UCT within a fixed time limit (Figure 4). Increasing the number of iterations provides more opportunity for improvement. dUCT-v1 reached iteration counts over twice that achieved by UCT. dUCT-v2 and eUCT were significantly better in solving more molecules than UCT, and all enhancements found more routes per molecule across all time constraints.

**Table 3:** Primary Testing Using AiZynthFinder 4.3 with the 50K ChEMBL Dataset^a^

method	time limit (s)	solve %	routes per molecule	avg no. of iterations
UCT	150	93.4	304.4	1487.7
eUCT	150	94.6	315.5	1633.8
dUCT-v1	150	91.4	402.1	4076.8
dUCT-v2	150	95.0	352.1	1855.4
UCT	100	91.4	204.2	1000.6
eUCT	100	92.1	210.7	1106.5
dUCT-v1	100	89.7	288.5	3173.3
dUCT-v2	100	92.9	239.8	1260.0
UCT	60	89.2	141.5	691.0
eUCT	60	90.0	148.3	729.8
dUCT-v1	60	87.6	198.4	2387.2
dUCT-v2	60	91.0	165.0	865.1

a eUCT and two variants of dUCT: v1 and v2, were run against the unmodified UCT for different time constraints using the MolBloom stock.

**Figure 4:**
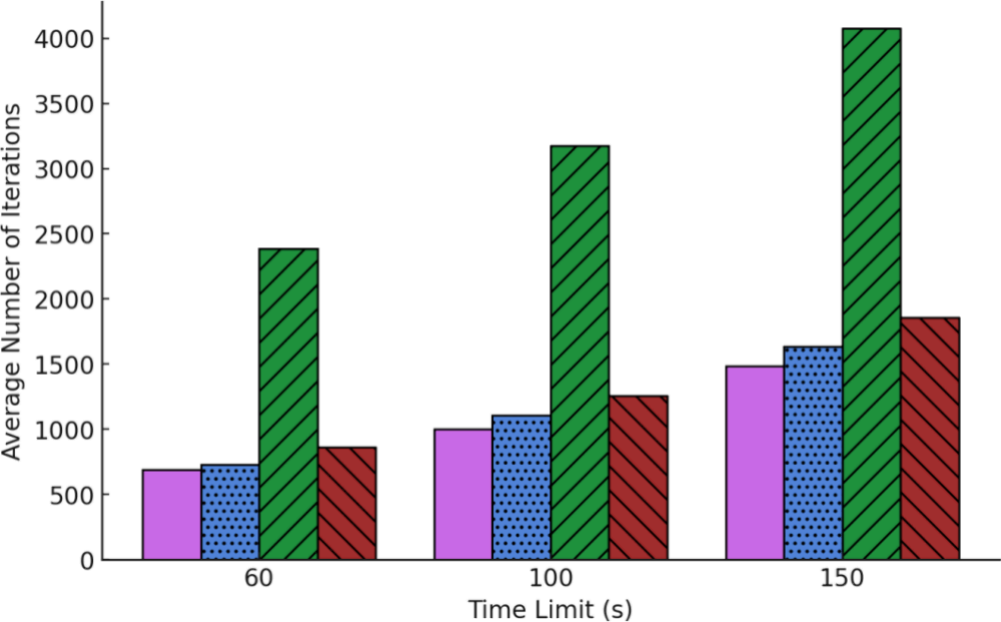
Number of MCTS iterations achieved in the three tested time constraints for the following enhancement types. **UCT**: pink-solid, **eUCT**: blue-stipple, **dUCT-v1**: green-upward hashing, **dUCT-v2**: red-downward hashing. Results obtained on the 50k-ChEMBL data set using the Molbloom stock.

PaRoutes is a framework designed for benchmarking retrosynthesis route predictions.^38^ Our enhancements to MCTS improve computational efficiency relative to the methods reported in the PaRoutes framework. At 100 iterations, our approach achieves an average search time per molecule of approximately 25–30 s, substantially lower than the approximately 300 s reported from PaRoutes. PaRoutes tested their MCTS using 500 iterations. Scaling the search to 1000 iterations, standard UCT required roughly 255 to 260 s per molecule, whereas the dUCT variants reduced this to between 99 and 170 s, allowing nearly twice the number of iterations within roughly half the computational clock time. At 5000 iterations, while UCT required over 1100 s per molecule, dUCT maintained a 40 to 50% reduction in search time, achieving an average of roughly 640 s. This improved efficiency not only reduces the overall computational cost but also allows a more thorough exploration of the search space, yielding in a higher average number of routes per molecule.

The dUCT variants outperform the alternative methods in the PaRoutes framework, Retro* and DFPN, in terms of computational clock time, while maintaining or improving the ratio of solved molecules. For 1000 iterations, dUCT-v1 achieved an average time per-molecule of 100 s, whereas Retro* typically required around 300 s under similar conditions. Retro* minimizes one-step model calls to reduce computational expenditure and memory usage but thus reduces search space coverage. DFPN converged more slowly and solved a smaller proportion of the targets.

DFPN casts retrosynthesis to an AND/OR problem using a graphical representation of molecules (OR-nodes) and reactions (AND-nodes). For each OR-node, a proof number is used to estimate how many child reactions must be found for the molecule to be solvable. Each AND-node’s proof number is the sum of its precursors’ proof numbers. The leaf node with the smallest proof number is expanded, followed by a depth-first traversal. The strength of DFPN stems from its pruning of unpromising search space through ‘proof metrics’. However, it typically finds fewer alternative routes, thus limiting route diversity, and contains no exploration heuristic or rollout, which can make it slower to converge on more difficult targets.

Retro* adapts the classic A* search by classifying the problem as an AND/OR graph with a learnt cost heuristic for each molecule. Child reactions are expanded according to a fixed template set, and the search is guided by a best-first traversal of the lowest costing reactions. This allows Retro* to locate high quality routes to a target in fewer node expansions. A limitation to Retro* is its reliance on the accuracy of the cost heuristic. Additionally, Retro* typically returns a single best solution over an array of alternative routes.

In contrast, AiZynthFinder relies on the balance of exploration and exploitation through MCTS. The rollout heuristic gives it an advantage over DFPN as it can balance depth and breadth more efficiently. Compared with Retro*, MCTS does not rely on a learnt cost function, and instead uses neural-guided simulations to estimate a node’s value. This can be more robust depending on the quality of the heuristic and will provide more alternative routes to a target. However, a vanilla MCTS requires tuning of iteration and exploration constants, which have a huge impact on the algorithm’s performance; a weakness of MCTS which is specifically targeted by our enhancements.

Direct comparisons with other approaches are difficult and thus the preceding discussion of relative performance is indicative rather than definitive. Neither 3N-MCTS nor SYNTHIA^39^ are open-source. They have been previously evaluated on in-house libraries of compounds. 3N-MCTS achieved a 95% solve proportion on 497 molecules with an average of 13 s per molecule. Best First Search (BFS) using a SMILES policy obtained a 56% solve proportion, taking an average of 420 s per molecule. SYNTHIA uses an extensive reaction rule database hand-encoded by expert synthetic chemists. As a rule-based method, it is template-free and uses different selection mechanisms than UCT. The SYNTHIA Full Retro API is available through request and reports a throughput of 50 molecules per hour.

The preceding discussion gives a statistical analysis of the value of the enhancements. We now turn to an illustrative example: almotriptan, a molecule used as a medication for migraines. It belongs to a class of compounds known as triptans, which are serotonin receptor agonists. It contains a sulfonamide group which is essential for its biological activity. Almotriptan was used as an exemplar in a previous retrosynthesis study^40^ using Chematica, which is commercially available as SYNTHIA.^39^ A graph-based algorithm with shared information across targets was used to generate libraries of compounds or isotopically labeled molecules. The algorithm helps to improve the synthesis of multiple targets simultaneously. This method was used to design synthetic plans for isotopically labeled variants of almotriptan.

The dUCT-v2 enhancement was tested against UCT using the ZINC stock. The number of routes, steps, and several sustainability metrics were taken for comparison. For each step in a route, the solvent, temperature, stoichiometry, element sustainability, atom economy and safety, were assigned scores between 1 and 3 (1 being the most sustainable) based-on the predicted reaction conditions.^41^ For every route, the average score across all steps was taken for each metric and summed to a total sustainability score for each route shown in Table 4. Routes 1 through 4 were found by both UCT and dUCT-v2, route 5^ was found solely by UCT, and routes 6* through 9* were found solely by dUCT-v2.

**Table 4:**
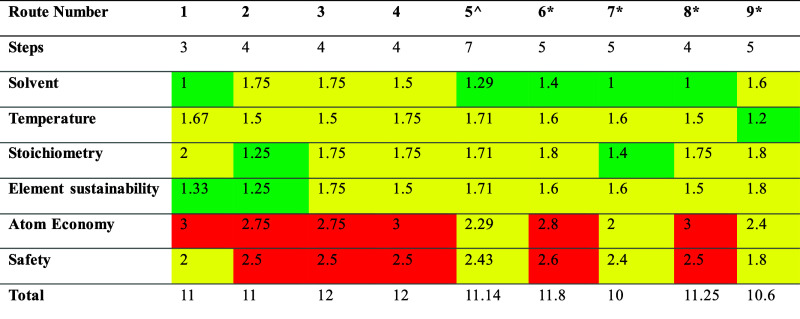
Sustainability Metrics for Ten Routes to Almotriptan Using the ZINC Stock File with a Time Limit of 100 s^a^

a Color-coding from condition prediction implemented in AI4Green. ^routes generated solely by UCT, *routes generated solely by dUCT-v2.

Each step in a route was considered in SciFinder^42^ to assess feasibility. Several common steps have literature precedent, such as the cyclization reaction to form an indole through the hydrazine reacting with the carbonyl.^43^ Common starting materials across the routes include 1,4-dibromobutane, *p*-fluorobenzoic acid, nitromethane and 1,2-dichloroethane. We observed better sustainability scores on the routes generated by dUCT-v2 compared to UCT. The average route score for all eight dUCT-v2 routes was 11.2, compared to 11.4 across UCT’s five routes. The four routes unique to dUCT-v2 averaged a sustainability score of 10.9. dUCT-v2 found the most sustainable route (7*) with a route score of 10 (Figure 5), and the second-best route (9*) with a score of 10.6. These were the only two routes which achieved scores below 11, and both contained five steps.

**Figure 5:**
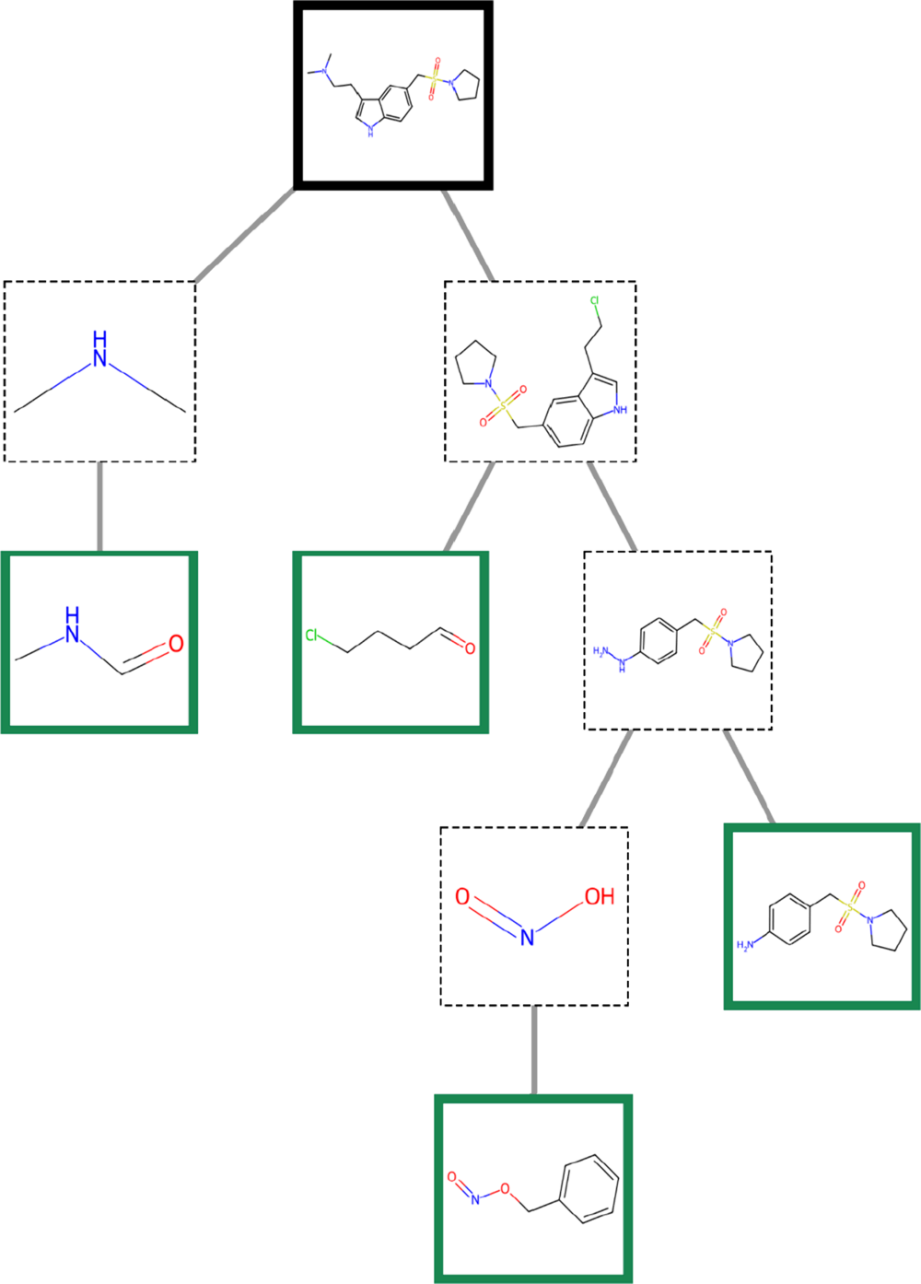
Retrosynthesis route for almotriptan, route 7* from Table 4, taken from https://ai4green.app. Target molecule in black, stock molecules in green.

## Conclusions

In this work, we proposed two MCTS enhancements, eUCT and dUCT, and tested them on the retrosynthesis program, AiZynthFinder, against the unmodified UCT. The enhancements were assessed with time-based and iteration-based controls, with two variations of the latter enhancement considered. dUCT almost always solved more molecules than UCT (Table 1), likewise for dUCT-v2 in Table 2. Tables 1 and 2 also show the decrease in computational clock-time achieved by dUCT-v1. eUCT gave more modest time reductions than the other enhancements. However, eUCT did not improve the proportion of solved molecules. Both dUCT-v2 and eUCT achieved higher solve rates for time-based testing and all enhancements showed improvements in the number of routes to a target molecule (Table 3). dUCT-v1 achieved the largest uplift against the default for the number of routes to the target, with at least a 30% increase across all time constraints. Therefore, dUCT-v1 yielded the highest RCS for all enhancements (Figure 3). Furthermore, for a given time, all enhancements reached larger iteration counts than UCT. The most impressive iteration increase was once again from dUCT-v1, which performed more than twice the number of iterations than UCT (Figure 4). The number of iterations is positively correlated to the number of solved molecules, and enhancements which allow more iterations will be beneficial for retrosynthesis. Reducing the clock-time directly reduces the computational carbon footprint. dUCT-v2 is the most effective at solving a random molecule for all time constraints and iteration values, and dUCT-v1 is the most useful for finding routes to a molecule which is solvable, within a given time. For an exemplar target molecule, almotriptan, dUCT-v2 found more routes than UCT (Table 4) and only dUCT-v2 found the two most sustainable routes. We conclude that our MCTS enhancements significantly improve the number of routes found per molecule, the computational clock-time to solve a molecule, and percentage of solved targets.

Future work will involve integrating sustainability metrics earlier into the process. Our current method estimates sustainability features post hoc, but it will be useful for the sustainability metrics to be used as features in the neural network template generation. This will allow suggested routes to have sustainable chemistry directly integrated into workflow. We will explore combining our enhancements with others and employ data-driven approaches to find how one might do this optimally.

## Data Availability

AI4Green is open-source and released under the AGPL-3.0 license. Full source code, installation instructions, and links to our video tutorials and user guides can be found at https://github.com/AI4Green/AI4Green. Code and associated data for the MCTS enhancements can be found at https://github.com/AI4Green/retrosynthesis-api. The full list of molecules tested in our experiments can be found at 10.6084/m9.figshare.28751012.v1.

## Abbreviations

CASP: Computer-Assisted Synthesis Planning
ELN: electronic lab notebook
eUCT: enhanced UCT
JSON: JavaScript Object Notation
LHASA: Logic and Heuristics Applied to Synthetic Analysis
MCTS: Monte Carlo tree search
RCS: rate-correct score
SMILES: Simplified Molecular-Input Line-Entry System
UCT: Upper Confidence bounds applied to Trees
USPTO: U.S. Patent and Trademark Office
